# Mutation of the Polyproline Sequence in CD3ε Evidences TCR Signaling Requirements for Differentiation and Function of Pro-Inflammatory Tγδ17 Cells

**DOI:** 10.3389/fimmu.2022.799919

**Published:** 2022-03-31

**Authors:** Aldo Borroto, Balbino Alarcón, María N. Navarro

**Affiliations:** Interactions with the Environment Program, Centro Biología Molecular Severo Ochoa, Consejo Superior de Investigaciones Científicas, Universidad Autónoma de Madrid, Madrid, Spain

**Keywords:** TCR signaling, Nck, Tγδ17, TCRgammadelta differentiation, IL-17, imiquimod, psoriasis

## Abstract

Tγδ17 cells have emerged as a key population in the development of inflammatory and autoimmune conditions such as psoriasis. Thus, the therapeutic intervention of Tγδ17 cells can exert protective effects in this type of pathologies. Tγδ cells commit to IL-17 production during thymus development, and upon immune challenge, additional extrathymic signals induce the differentiation of uncommitted Tγδ cells into Tγδ17 effector cells. Despite the interest in Tγδ17 cells during the past 20 years, the role of TCR signaling in the generation and function of Tγδ17 cells has not been completely elucidated. While some studies point to the notion that Tγδ17 differentiation requires weak or no TCR signaling, other works suggest that Tγδ17 require the participation of specific kinases and adaptor molecules downstream of the TCR. Here we have examined the differentiation and pathogenic function of Tγδ17 cells in “knockin” mice bearing conservative mutations in the CD3ε polyproline rich sequence (KI-PRS) with attenuated TCR signaling due to lack of binding of the essential adaptor Nck. KI-PRS mice presented decreased frequency and numbers of Tγδ17 cells in adult thymus and lymph nodes. In the Imiquimod model of skin inflammation, KI-PRS presented attenuated skin inflammation parameters compared to wild-type littermates. Moreover, the generation, expansion and effector function Tγδ17 cells were impaired in KI-PRS mice upon Imiquimod challenge. Thus, we conclude that an intact CD3ε-PRS sequence is required for optimal differentiation and pathogenic function of Tγδ17 cells. These data open new opportunities for therapeutic targeting of specific TCR downstream effectors for treatment of Tγδ17-mediated diseases.

## Introduction

In the past 20 years, TCRgamma delta cells (Tγδ) have emerged as an essential lymphoid population in the defense against pathogen infections, with critical roles in the development of pathological conditions such as autoimmune diseases and cancer (1–3). One key feature of Tγδ cells is their rapid response to immune challenges, characterized by the secretion of large amounts of interleukin 17 (IL-17) or interferon gamma (IFNγ) that are produced by distinct subpopulations of Tγδ cells (Tγδ17 and Tγδ−IFNγ, respectively). Tγδ17 cells provide protection against bacteria and fungi and they are essential for immune response against specific pathogens (i.e., *E. coli*, *S. aureus* or *C. albicans*) (4–6). Moreover, Tγδ17 are often the first responders and main source of IL-17 in models of inflammatory and autoimmune diseases such as psoriasis or multiple sclerosis, creating a pro-inflammatory milieu that conditions the adaptive immune response (2, 7). In comparison to the Tαβ lineage that requires antigen encounter followed by 5-7 days of differentiation to acquire effector functions, Tγδ cells commit to IL-17 production during thymic development. Additionally, there are extrathymic signals such as IL-23 and IL-1β that induce the differentiation of naïve Tγδ cells into Tγδ17 effector cells upon immune challenge (8, 9). In addition to the cytokine production profile, several studies have contributed to the identification of specific markers to the define Tγδ17 subpopulation. These studies have determined that Tγδ17 cells express the Th17 master transcription factor RORγt, and they are characterized by the expression of high levels of the cell surface marker CD44, and lack of CD27 and CD45RB expression (CD44^hi^CD27^neg^CD45RB^neg^) (10, 11).

Tγδ cells are positioned in boundary between innate and adaptive immune response, and several research groups have undertaken the task of determining the role of TCR signaling in the intrathymic commitment of Tγδ cells. Overall, different works suggest that Tγδ differentiation require a quantitatively different TCR signaling: strong TCR signaling leads to commitment towards IFNγ secretion, while Tγδ17 cells require weak or no TCR signals. In this context, some studies suggested that the Tγδ17 lineage programming occurs before TCRγδ rearrangements (12, 13). Other work using transgenic TCRγδ receptors recognizing T10 and T22 antigens showed that antigen-experienced cells made IFNγ, while antigen-naïve cells were diverted towards IL-17-producing phenotype (14). A study that identified Skint-1 as a thymic epithelial determinant in dendritic epidermal Tγδ cells (DETCs) suggested that TCR ligation switched down the IL-17-differentiation program (15), and TCR triggering using an anti-TCRγδ antibody in fetal organ thymic cultures reduced the generation of Tγδ17 cells (16). In a model of attenuated TCR signaling, CD3γ and CD3δ double haploinsufficient adult mice had normal frequencies of Tγδ17 cells (17). However, the complete picture is likely to be more complicated. Tγδ17 cells constitutively express markers that are associated with TCR activation (i.e., high levels of TCRγδ, CD44, CD127, IL-1R, CCR6) (18–20). Furthermore, several studies in kinase-deficient animals that result in attenuated TCR signaling reported a specific impairment of Tγδ17 cell differentiation. For example, the B lymphoid kinase (Blk), a B cell-specific member of the Src family of protein tyrosine kinases, was specifically required for the development of Tγδ17 cells (21). SKG mice bearing a hypomorphic mutation in the ZAP-70 tyrosine kinase that resulted in attenuated TCR signaling, displayed a pronounced deficiency of Tγδ17 cells (22). Moreover, the fine tune regulation of the activation of the tyrosine kinase Syk regulated Tγδ17 differentiation (23, 24). All together, these data point to the idea that differentiation of Tγδ17 cells may involve weak TCR signals, but also the participation of specific signaling molecules.

Tγδ17 cells provide protection against specific pathogens, but their effector function is closely linked to the development of autoimmune and inflammatory diseases such as psoriasis (2). Psoriasis is a chronic, relapsing/remitting inflammatory skin condition that affects 1-5% of the world population, characterized by red, scaly and itchy plaques in the skin with a number of associated comorbidities (25, 26). Psoriasis is a complex multifactorial condition in which the excessive production of IL-17 is key driver of psoriasis pathogenesis (27, 28). Tγδ17 cells are required for the development of Imiquimod (IMQ) skin inflammation model (29, 30). This model is based on the topical application of a cream containing IMQ, a TLR7/8 ligand that induces skin lesions. The histological analysis of these lesions by hematoxylin/eosin staining shows features that resemble those found in human psoriasis such as epidermal thickening (acanthosis) and leucocyte infiltration (31), and induces *de novo* generation and expansion of Tγδ17 cells (8). Tγδ17 cells expand in the LN, and then migrate to the inflamed skin (30, 32, 33), where the development of the skin lesions in the IMQ model partially depends on the secretion of IL-17A by Tγδ17 cells (29, 34, 35). Of note, the requirements of TCRγδ signaling for Tγδ17 effector function has not been extensively addressed, but some work suggest that TCRγδ signaling is required to establish a long-lived memory Tγδ17 population that mediate an exacerbated response upon a second Imiquimod challenge (36).

Overall, the role of TCR signaling during Tγδ17 development and pathogenic function has not been completely elucidated. In this context, previous work in “knockin” mice bearing conservative mutations of the two central prolines in the CD3ε polyproline rich sequence (KI-PRS, PxxP to AxxA point mutations) with attenuated TCR signaling due to lack of binding the essential adaptor “non-catalytic region of tyrosine kinase” (Nck), showed impaired differentiation and effector function of TCRαβ cells (37, 38). Further work found both decreased frequency and numbers of cells bearing the TCRγδ-Vγ2 variable region in the adult thymus (39) [Vγ2 following Garman´s nomenclature (40), called Vγ4 by Heiling and Tonegawa (41)]. Interestingly, Vγ2 rearrangements are particularly frequent among Tγδ17 cells (42). Taken together, the data suggest that the CD3ε-PRS-dependent TCR signaling might be required for Tγδ17 commitment. Here, we have determined that KI-PRS mice presented decreased frequency and numbers of Tγδ17 cells in adult thymus and lymph nodes. We have addressed the pathogenic function of Tγδ17 cells in the IMQ model of skin inflammation. KI-PRS displayed attenuated skin inflammation compared to wild-type littermates. Moreover, the expansion and effector function of Tγδ17 cell were impaired in KI-PRS mice. Overall, we conclude that an intact CD3ε-PRS sequence is required for both optimal differentiation and pathogenic function of Tγδ17 cells, revealing a specific TCR signaling dependence for development and function of these pro-inflammatory cells. Our results point to the notion that the diversity of signaling outcomes emanating from the TCR may be modulated by the composition of the TCR signalosome and thus, small changes in the configuration of TCR downstream effectors may influence signaling outcomes such as Tγδ differentiation. These data open new opportunities for therapeutic intervention of specific TCR signaling pathways for the treatment of Tγδ17-mediated diseases.

## Materials and Methods

### Mice

Knock-in mice bearing the PxxP to AxxA double mutation in the polyproline sequence of CD3ε (KI-PRS) have been previously described (37). The experiments were performed in homozygous littermates for the WT or knock-in alleles. Mice were maintained under specific pathogen–free (SPF) conditions at the animal facility of the Centro de Biología Molecular Severo Ochoa. Mice breeding and procedures were performed in accordance with national and institutional guidelines for animal care (EU Directive 2010/63/EU for the protection of animal used for scientific purposes). The experimental procedures were approved by the Director General de Medio Ambiente de la Comunidad de Madrid (Approval reference: PROEX 296-7-21).

### Flow Cytometry

Thymuses and lymph nodes (LN) from KI-PRS and WT mice were harvested, pooled and mechanically disaggregated. For dead cell exclusion, cells were incubated with Ghost Dye-Red780 following manufacturer´s instructions (Tonbo Biosciences), supplemented with Fc block (ref. 553142; BD Biosciences) for 30min/ice prior to antibody staining. For surface staining, cells were washed once in staining solution (PBS 1% bovine serum albumin) and incubated for 20min/ice following manufacturer´s suggested antibody dilutions in staining solution. In some stainings, biotin-coupled antibodies followed by fluorochrome-coupled streptavidin were used. For measurement of IL-17A production *ex vivo*, cells were stimulated with Phorbol 12, 13-Dibutyrate (PDBu, 20 ng/ml), Ionomycin (Io, 0.5ng/ml) for 6h in presence of GolgiPlug (BD Biosciences) for the last 4h, or Golgi-Plug alone, and processed for detection of intracellular cytokines by flow cytometry. For intracellular staining of IL-17A production, after cell surface staining cells were fixed for 20 min/RT (IC Fixation buffer; Thermo Fisher), and incubated with anti-IL-17A diluted in Permeabilization buffer (Thermo Fisher)), for 30min/RT, following manufacturer´s instructions. RORγt intracellular staining was performed using FoxP3/Transcription Factor Staining set (Thermo Fisher) following manufacturer´s instructions. Countbright absolute counting beads (ref. C36950; Invitrogen) were added before processing the samples for flow cytometry analysis to determine absolute cell numbers. Samples were acquired on a FACSCanto II flow cytometer with DIVA software and analyzed with FlowJo software (Tree Star). Cells were gated according to their forward scatter and side scatter profile, and dead cells excluded based on their staining with the viability dye. Graphad Prism v.6 was used for statistical analysis. Statistical analysis was performed using Mann-Whitney t-test.

### Antibodies and Other Reagents

The following fluorochrome-coupled versions of these antibodies were used in this study. The number in brackets indicates the manufacturer´s reference.

*Purchased from BD Pharmigen*: PE-anti-CD3ε (553064), PerCP-C5.5-anti-IL-17A (560666), FITC-anti-CD122 (553361), BV605-anti-Vγ2 TCR (742310), Biotin-anti-CD4 (553045), PE-anti-CD45.2 (560695), FITC-anti-Ly-6C (553104), PerCP-C5.5- anti-CD64 (561194), PE-Cy7-anti-Ly-6G (560601), APC-anti-CD11c (550261), Biotin-anti-CD11b (553309), FITC-anti-CD24 (553261), BV605-Streptavidin (563260) and PE-C7-Streptavidin (557598). *From eBioscience/Invitrogen:* PerCP-eFluor710-anti-TCRγ/δ (46-5711-82), APC-anti-CD27 (17-0271-82), PE-Cy7-anti-TCR Vγ2 (25-5828-82), APC-anti-RORγt (17-6988-82), and Biotin-anti-CD8a (13-0081-85). *From Biolegend*: BV421-anti-CD44 (103040), BV421-anti-TCRγδ (118119), PE/Cy7-anti-CD27 (124216), APC-anti-CD45RB (103320), APC-anti-CD73 (127210) and BV421-Streptavidin (405225). *From Miltenyi Biotec*: APC-anti-IFNγ (130-120-805).

### Imiquimod Skin Inflammation Model

KI-PRS and WT littermates mice were treated with 5% Imiquimod on shaved and depilated back and ear skin for 7 days (50 mg/day; Aldara; Meda Pharma), or left untreated. At the experimental endpoint, flow cytometry was performed on mouse ears and skin draining LN. Immunohistochemistry analyses were carried out on mouse back skin. Skin draining LN (cervical, axillary, brachial and inguinal) were harvested, pooled and mechanically disaggregated for flow cytometry analysis. Ears were split in two halves, cut into pieces and digested for 45min/37°C in RPMI containing Liberase TM (83 μg/ml; Roche), DNase I (100 μg/ml; Roche) and Collagenase IV (0.5 mg/ml; Sigma). Undigested skin pieces were further subjected to tissue disruption using 7 mm stainless steel beads (Qiagen) and a TissueLyser LT (20 oscilations/5 min; Qiagen). Samples of skin from mice’s backs were rapidly immersed fixed in 4% paraformaldehyde and embedded in paraffin. For the histological study, skin slices (4-5 μm thick taken 200μm apart) were stained with hematoxylin and eosin (H&E). For IHC staining, skin sections were deparaffinized, boiled in antigen retrieval solution (10mM sodium citrate, 0,05% Tween 20, pH6). Slides were developed with DAB substrate (Dako K3468) and then counterstained with Mayer’s Hematoxylin. Images were captures using an Olympus microscope BX41, 10x objective, with an Olympus camera DP-70 (Olympus Denmark A/S). Epidermal thickness was quantified in different skin sections (8 sections per mouse, 32 measures per section), using ImageJ software.

### Statistical Analysis Section

All datasets were subjected to D’Agostino & Pearson omnibus normality test to determine Gaussian distribution. The datasets did not pass normality test and accordingly, the statistical significances were obtained using the non-parametric Mann-Whitney two-tailed t-test. Graphad Prism v.6 was used for statistical analysis.

## Results

### CD3ε-PRS Sequence Is Required for Tγδ17 Differentiation in Adult Mice

**Figure 1:**
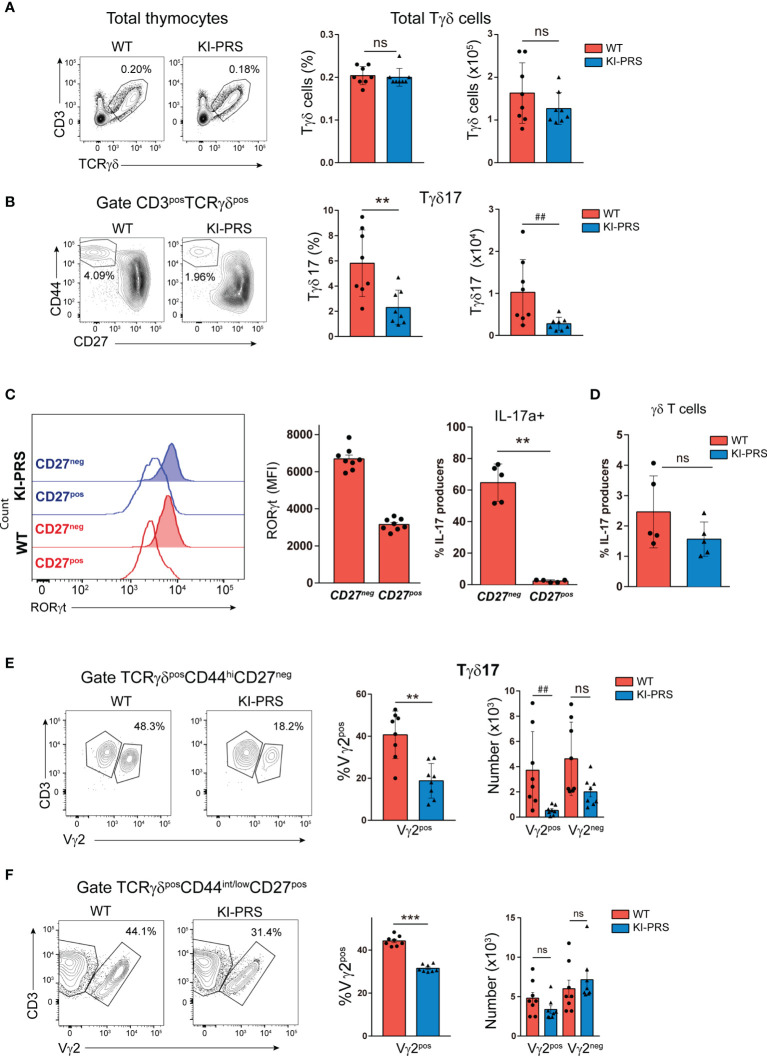
Mutations in the polyproline sequence of CD3ε impair Tγδ17 commitment in the thymus. Thymuses from 8-week old KI-PRS and wild-type (WT) littermates were harvested and processed for flow cytometry analysis of Tγδ cell subsets. **(A)** Representative dot plots show CD3 and TCRγδ expression in total thymocytes. Graphs represent the frequency (*left graph*) and absolute cell number (*right graph*) of total Tγδ cells gated as CD3^pos^TCRγδ^pos^. **(B)** Representative dot plots of CD44 and CD27 expression among Tγδ cells. Graphs represent the frequency (*left*, **p-value= 0.0047) and absolute cell number (*right*, ^##^p-value= 0.0070) of Tγδ17 cells, gated as CD3^pos^TCRγδ^pos^CD44^hi^CD27^neg^. **(C)** Representative histograms show the expression of the transcription factor RORγt among Tγδ^pos^CD44^hi^CD27^neg^ cells (Tγδ17) and Tγδ^pos^CD44^int/low^CD27^pos^ cell (uncommitted Tγδ). Graph represents the mean of fluorescence intensity (MFI) of RORγt among the indicated populations. Analysis of IL-17A production. Total thymocyte suspensions were stimulated with PDBu/Io for 6h in presence of Golgi-Plug, and IL-17A production was determined by flow cytometry. Graph represents the frequency of IL-17A-producers among CD44^hi^CD27^neg^ and CD44^int/low^CD27^pos^ Tγδ cells. **(D)** Frequency of γδT cells IL17a producers. **(E)** Representative dot plots show Vγ2 expression among Tγδ17 cells (gated as TCRγδ^pos^CD44^hi^CD27^neg^). Graphs represent the frequency (*left*, **p-value= 0.0019) and absolute cell number (*right*, ^##^p-value= 0.0019) of Vγ2^pos^ and Vγ2^neg^ cells among Tγδ17 cells. **(F)** Representative dot plots show Vγ2 expression among uncommitted Tγδ cells (gated as TCRγδ^pos^CD44^int/low^CD27^pos^). Graphs represent the frequency (*left*, ***p-value= 0.0002) and absolute cell number (*right*) of Vγ2^pos^ and Vγ2^neg^ cells among uncommitted Tγδ cells. The inset numbers in representative dot plots represent the percentage of cells within the indicated gate. All graphs represent mean ± sd of n=7-8 mice of each genotype. Statistical analysis was performed using Mann-Whitney t-test. ns, non significant. Data are representative of 2 independent experiments.

To determine if the TCR signaling emanating from the polyproline rich sequence of CD3ε (CD3ε-PRS) was required for commitment towards the Tγδ17 lineage in the adult thymus, we analyzed TCRγδ subpopulations in “knockin” mice bearing two conservative mutations in the CD3ε-PRS (PxxP to AxxA change, KI-PRS mice) (37–39). Total Tγδ cells frequency and absolute cell number in KI-PRS adult mice were not significantly different from those of wild-type littermates (WT) (**Figure 1A**). However, the specific analysis of mature Tγδ17 cells (defined as TCRγδ^pos^CD44^hi^CD27^neg^ cells) showed a marked decrease in frequency and absolute cell number of those cells in KI-PRS mice compared to WT littermates (**Figure 1B**). The TCRγδ^pos^CD44^hi^CD27^neg^ population was confirmed to identify the Tγδ17 lineage because this population and not the CD27^pos^ one is RORγt+ and expresses intracellular IL-17A (**Figure 1C**). Additionally, we compared IL-17A production by thymic Tγδ cells cells from WT and KI-PRS mice in response to stimulation with phorbol esters and ionomycin. These experiments showed that Tγδ cells in the thymus are less competent to produce IL-17A in KI-PRS mice than their WT counterparts, although in this case the difference did not reach significance (**Figure 1D**).All together, the CD44 and CD27, RORγt and IL-17A expression data showed that KI-PRS mice had a lower number of mature Tγδ17 cells in the thymus. Rearrangements involving the Vγ2 variable region are particularly abundant among Tγδ17 cells (42). Our previous studies found that the frequency and number of Vγ2 cells among total Tγδ cells were reduced KI-PRS mice (39). Thus, we next determined whether Vγ2 usage among mature Tγδ17 cells in KI-PRS mice. The frequency and absolute cell number of Tγδ17-Vγ2^pos^ cells were strongly diminished in KI-PRS mice compared to WT (**Figure 1E**), and we observed a slight but non-significant reduction in the number of Tγδ17-Vγ2^neg^ cells. We also found an underrepresentation of Vγ2 usage among Tγδ subsets that were not committed to Tγδ17 lineage (TCRγδ^pos^CD44^int/low^CD27^pos^) (**Figure 1F**), suggesting that the CD3ε-PRS mutation reduces the differentiation Tγδ cells expressing Vγ2, regardless their commitment towards the Tγδ17 lineage. Nonetheless, the overall result shows that an intact CD3ε-PRS sequence is required for commitment towards the Tγδ17 lineage in the adult thymus.

**Figure 2:**
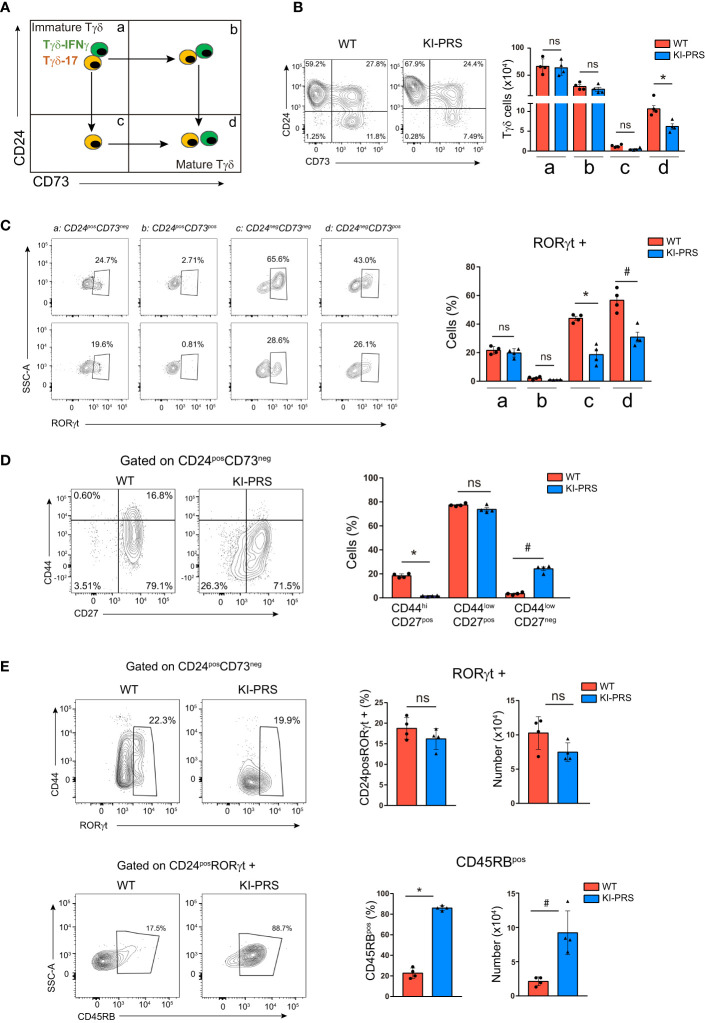
Impairment in Tγδ17 commitment in KI-PRS mice occurs in immature CD24^pos^CD73^neg^ progenitors. Thymuses from 8-week old KI-PRS and wild-type (WT) littermates were harvested and processed for flow cytometry analysis of Tγδ cell subsets. **(A)** Schematic representation of the developmental stages of Tγδ progenitors based on CD24 and CD73 expression. **(B)** Representative dot plots show CD24 against CD73 expression among Tγδ thymocytes (gated as CD3^pos^TCRγδ^pos^). Graph represents the absolute cell number in the quadrant regions shown in 2a (*p-value= 0.0286). **(C)** representative dot plots show RORγt expression in the different stages of maturation. Graph represents the frequency of RORγt cells in quadrant a,b,c,d (*p-value= 0.0286, ^#^p-value= 0.0286) **(D)** Representative dot plots show CD44 and CD27 expression among immature Tγδ progenitors (gated as CD3^pos^TCRγδ^pos^CD24^pos^CD73^neg^). Graph represents the frequency of cells in the indicated gates (*p-value= 0.0286, ^#^p-value= 0.0286). **(E)** Top, representative dot plots show CD44 against RORγt expression among immature Tγδ progenitors (gated as CD3^pos^TCRγδ^pos^CD24^pos^CD73^neg^). Graphs represent the frequency and absolute cell number of RORγt-expressing cells among immature Tγδ progenitors. Bottom, representative dot plots show CD45RB expression among RORγt-expressing immature progenitors (gated as CD3^pos^TCRγδ^pos^CD24^pos^CD73^neg^RORγt^pos^). Graphs represent the frequency and absolute cell number of CD45RB-expressing cells among RORγt-expressing immature progenitors (*p-value= 0.0286, ^#^p-value= 0.0286). All graphs represent mean ± sd of n=4 mice of each genotype. Statistical analysis was performed using Mann-Whitney t-test. ns, non significant. Data are representative of 2 independent experiments.

For a more detailed study of the developmental impairment in the commitment towards Tγδ17 lineage, we explored different stages of TCRγδ cell differentiation in the thymus following the expression of CD24 and CD73 markers. The immature Tγδ progenitors are defined as CD24^pos^CD73^neg^ (**Figure 2A**, stage a). From this stage, the Tγδ17 progenitors first down-regulate CD24 (CD24^neg^CD73^neg^, stage c), and finally up-regulate the expression CD73 before exiting the thymus as mature CD24^neg^CD73^pos^ Tγδ17 cells (**Figure 2A**, stage d). In contrast, Tγδ-IFNγ CD24^pos^CD73^neg^ progenitors first up-regulate CD73 (CD24^pos^CD73^pos^, stage b) and finally down-regulate CD24 (CD24^neg^CD73^pos^, stage d) (**Figure 2A**) (43, 44). The CD24 vs CD73 expression pattern was apparently normal in KI-PRS thymuses compared to WT littermates, with a slight decrease in the frequency of mature CD24^neg^CD73^pos^ Tγδ cells (**Figure 2B**). The absolute cell number of CD24^pos^CD73^neg^ immature precursors (stage a) was normal in KI-PRS mice, whilst a reduction in cell numbers of the last Tγδ17 differentiation stages (CD24^neg^CD73^pos^, stage d) was detected, suggesting that the Tγδ17 developmental impairment was occurring beyond the most immature stage (CD24^pos^CD73^neg^) (**Figure 2B**). Although we did not find a significant decrease in the intermediate maturation populations (stages b and c, **Figure 2A**), we carried out a intracellular staining with RORγt in order to identify which of those intermediate stages is precursor of the late differentiation stage d. We found that CD24^neg^CD73^neg^ (stage c) cells contained abundant RORγt+ cells, whereas CD24^pos^CD73^pos^ (stage b) were basically depleted of RORγt+ cells. Those data suggest that in adult murine thymus the order of differentiation of Tγδ17 cells is stages a-c-d and does not seem to involve stage b. Although did not observe statistically significant differences in the percentage of intermediate CD24^neg^CD73^neg^ between WT and KI-PRS mice (stage c, **Figure 2B**), we did however find a significant difference in the percentage of stage c cells that were RORγt+. This suggest that the impairment in Tγδ17 cell maturation in the thymus occurring in KI-PRS mice is already occurring at the intermediate CD24^neg^CD73^neg^ (stage c) population.

Thus, we analyzed the CD24^pos^CD73^neg^ subpopulation for hallmarks of Tγδ17 differentiation such as CD44, CD27 and RORγt expression. The analysis of the CD44 vs CD27 expression pattern in immature progenitors showed an accumulation of cells with lower levels of both CD27 and CD44 in KI-PRS compared to WT thymocytes (**Figure 2D**). These results indicated that KI-PRS Tγδ17 progenitors have commenced the down-regulation of CD27 expression, but they fail to up-regulate CD44. Further analysis of RORγt expression in the CD24^pos^CD73^neg^ population showed that KI-PRS mice have slightly lower frequency and number of RORγt+ cells, although the data did not reach statistical significance (**Figure 2E**). These data suggest that PRS sequence was not essential for RORγt expression in immature Tγδ17 progenitors. However, the immature RORγt-expressing cells remained CD44low (**Figure 2E**). We also determined the expression of CD45RB in the immature Tγδ17 progenitors, as CD45RB expression is down-regulated during the differentiation of Tγδ17 cells (16). This analysis showed that immature CD24^pos^RORγt+ cells failed to down-regulate CD45RB in KI-PRS mice (**Figure 2E**). Regarding the differentiation of Tγδ-IFNγ progenitors, we did not detected differences in absolute cell number of the intermediate stage CD24^pos^CD73^pos^ (**Figure 2B**), suggesting that Tγδ-IFNγ development may not be affected in KI-PRS mice. Collectively, the results in **Figure 2** show that the TCR signals emanating from CD3ε-PRS are required in immature Tγδ17 progenitors to up-regulate the expression of CD44 and to down-regulate CD45RB.

**Figure 3:**
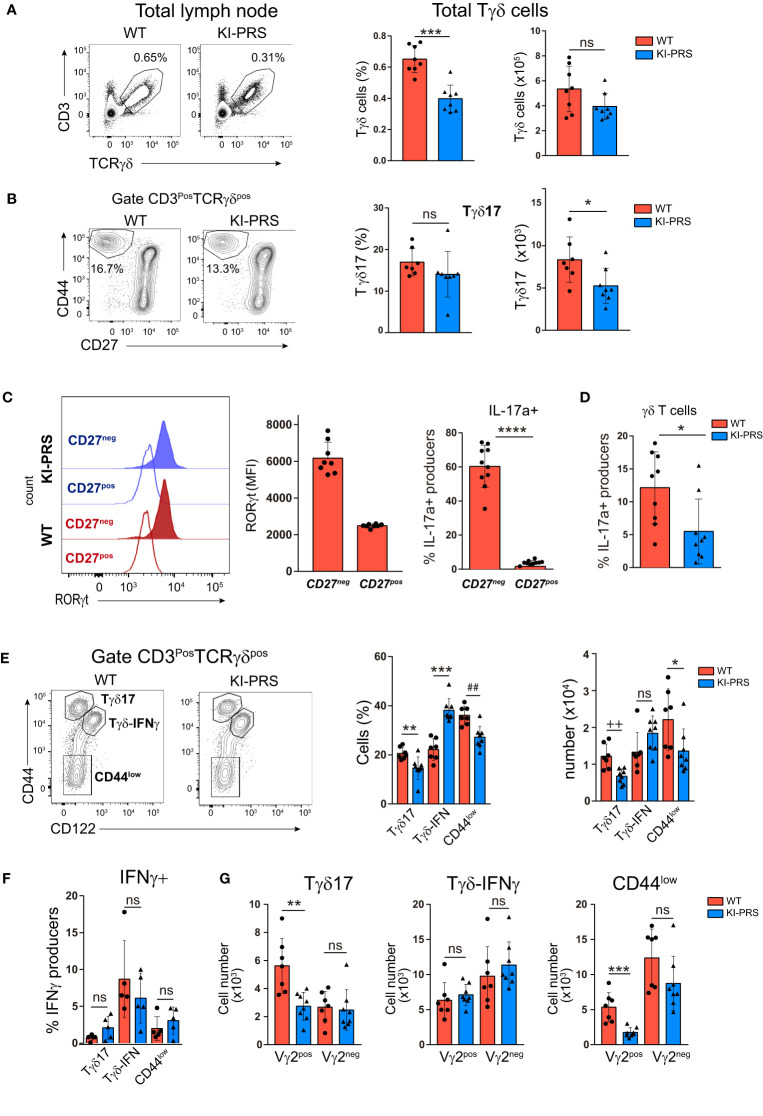
Intact CD3ε-PRS is required for Tγδ17 homeostasis in lymph nodes. Lymph nodes from 8-week old KI-PRS and WT littermates were harvested and processed for flow cytometry analysis of Tγδ cell subsets. **(A)** Representative dot plots show CD3 and TCRγδ expression in total lymph node cells. Graphs represent the frequency (*left*, ***p-value= 0.0003) and absolute cell number (*right*) of total Tγδ cells gated as CD3^pos^TCRγδ^pos^. **(B)** Representative dot plots of CD44 and CD27 expression among Tγδ cells. Graphs represent the frequency (*left*) and absolute cell number (*right*, *p-value= 0.0401) of Tγδ17 cells, gated as CD3^pos^TCRγδ^pos^CD44^hi^CD27^neg^. **(C)** Representative histograms show the expression of the transcription factor RORγt among Tγδ^pos^CD44^hi^CD27^neg^ cells (Tγδ17) and Tγδ^pos^CD44^int/low^CD27^pos^ cell (uncommitted Tγδ). Graph represents the mean of fluorescence intensity (MFI) of RORγt among the indicated populations.Analysis of IL-17A production. Lymph node cell suspensions were stimulated with PDBu/Io for 6h in presence of Golgi-Plug, and IL-17A production was determined by flow cytometry. Graph shows the frequency of IL-17A-producers among CD44^hi^CD27^neg^ and CD44^int/low^CD27^pos^ Tγδ cells ****P-value<0.0001. **(D)** Frequency of IL17 producers in γδT cells (*p-value: 0.0242) **(E)** Representative dot plots of CD44 and CD122 (IL-2Rβ) expression among Tγδ cells. Graphs represent the frequency (*left*, **p-value= 0.0059, ***p-value= 0.0003, ^##^p-value= 0.0037) and absolute cell number (*right*, ^++^p-value= 0.0037; *p-value= 0.0406) of Tγδ17 cells (gated as CD3^pos^TCRγδ^pos^CD44^hi^CD122^neg^), Tγδ-IFNγ (gated as CD3^pos^TCRγδ^pos^CD44^int^CD122^pos^) and uncommitted Tγδ cells (CD44^low^, gated as CD3^pos^TCRγδ^pos^CD44^low^CD122^neg^). **(F)** Analysis of IFNγ production. Lymph node cell suspensions were stimulated with PDBu/Io for 6h in presence of Golgi-Plug, and IL-17A production was determined by flow cytometry. Graph shows the frequency of IFNγ-producers among Tγδ17 cells, Tγδ-IFNγ and uncommitted Tγδ cells (CD44low), gated as in **(E)**. **(G)** Graphs represent the absolute cell number of Vγ2^pos^ and Vγ2^neg^ cells among Tγδ17 cells (**p-value= 0.0022), Tγδ-IFNγ and uncommitted Tγδ cells (***p-value= 0.0006), gated as in **(E)**. The inset numbers in representative dot plots represent the percentage of cells within the indicated gate. All graphs represent mean ± sd of n=7-8 mice of each genotype. Statistical analysis was performed using Mann-Whitney t-test (n=8 mice of each genotype). ns, notsignificant. Data are representative of 2 independent experiments.

Next, we investigated if the reduction of mature Tγδ17 cells observed in the thymus was maintained in the periphery of adult KI-PRS mice. The analysis of lymph nodes (LN) showed a reduction in percentage of total Tγδ cells compared to WT littermates, and a slight but non-significant decrease in cell numbers (**Figure 3A**). As previously found in the KI-PRS thymus, the frequency and absolute cell number of Tγδ17 cells in KI-PRS LN were reduced (**Figure 3B**), albeit this defect was not as prominent in LN as in the thymus (compare **Figures 1B**, **3B**) As shown in the thymus (**Figure 1C**) we found that the CD27neg population is the one that expresses the highest levels of RORγt and intracellular IL-17A (**Figure 3C**). As for thymic Tγδ17 cells (**Figure 1D**) we found that lymph node Tγδ17 cells from KI-PRS mice produced less IL-17A than their WT counterparts, but in this occasion the differences were statistically significant (**Figure 3D**). We also examined if the CD3ε-PRS mutation affected other Tγδ subsets in the LN. In particular, we analyzed the subpopulation of Tγδ-IFNγ producers, characterized by the expression of CD122 (IL-2β chain) and intermediate levels of CD44 (Tγδ-IFNγ: CD44^int^CD122^pos^) (14, 42), and the subpopulation of uncommitted Tγδ cells (Tγδ-CD44^low^: CD44^low^CD122^neg^) (**Figure 3E**). We found that both Tγδ17 and Tγδ^pos^CD44^low^ subsets were decreased both in percentage and absolute cell number (**Figure 3E**). By contrast, the Tγδ-IFNγ population was increased in frequency and unaltered in absolute cell number (**Figure 3E**). We also assessed IFNγ production in the lymph node Tγδ subpopulations, and determined that Tγδ-IFNγ (CD44^int^CD122^pos^) produced IFNγ while the Tγδ17 and Tγδ^pos^CD44^low^ subpopulations did not have the potential to secrete IFNγ (**Figure 3F**). Moreover, no significant differences in IFNγ production were detected in KI-PRS Tγδ-IFNγ cells compared to WT littermates, suggesting that an intact CD3ε-PRS was not required for maintenance of Tγδ-IFNγ cells in the lymph nodes. The analysis of Vγ2 usage among the three Tγδ subsets found a reduction in Vγ2^pos^ cells among Tγδ17 and the uncommitted Tγδ-CD44^low^ cells, whereas Vγ2 usage among the Tγδ-IFNγ population was not affected by the PRS mutation (**Figure 3G**). To summarize, **Figures 1**–**3** demonstrate that Tγδ17 differentiation was impaired in KI-PRS mice, while the development of Tγδ-IFNγ cells was not affected.

### Formation of IMQ-Induced Psoriatic-Like Lesions and Tγδ17 Skin Infiltration Are Ameliorated in KI-PRS Mice

**Figure 4:**
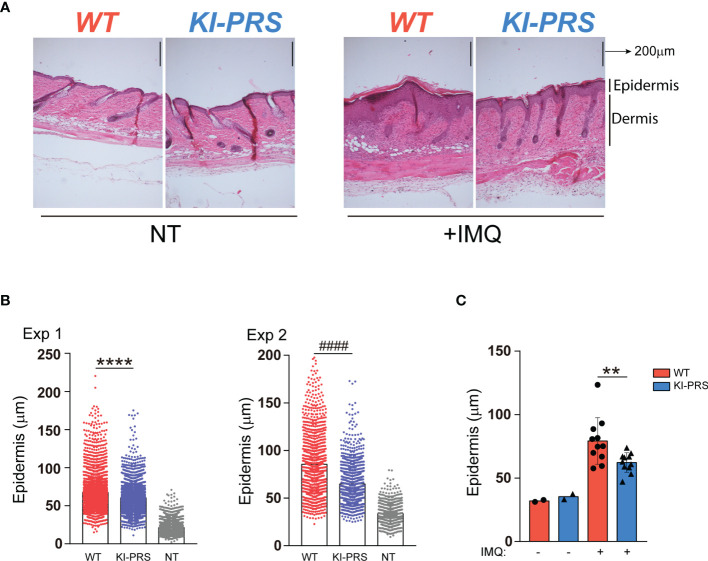
Formation of IMQ-induced psoriasis-like lesions is ameliorated in KI-PRS mice. KI-PRS and WT littermates were treated with Imiquimod (IMQ) for 7 days on ears and shaved backs, or left untreated (NT). On day 7, back skin sections were subjected to hematoxylin and eosin (H/E) staining for microscopy analysis. The thickness of the epidermal layer was measured at multiple sections and sites, randomly chosen in a blind manner. **(A)** Representative sections of H/E staining in the indicated conditions. Dermal and epidermal layers are indicated on the right. **(B)** Graphs represent all individual measurements of the epidermal layer thickness in 2 independent experiments. Each dot represents a single measure (8 sections per mouse, 32 measures per section). In experiment number 1, n= 5 treated mice from each genotype and n= 2 WT untreated mice were used (****p-value< 0.0001). In experiment 2, n= 6 treated mice from each genotype and n=4 untreated WT animals were used (^####^p-value< 0.0001). **(C)** Graph represents the epidermis thickness (mean ± sd). Each dot represents the thickness measurement per mouse (averaged value of 8 sections per mouse, 32 measures per section) from both experiments (n=11, **p-value= 0.0083). Statistical analysis was performed using Mann-Whitney t-test.

**Figure 5:**
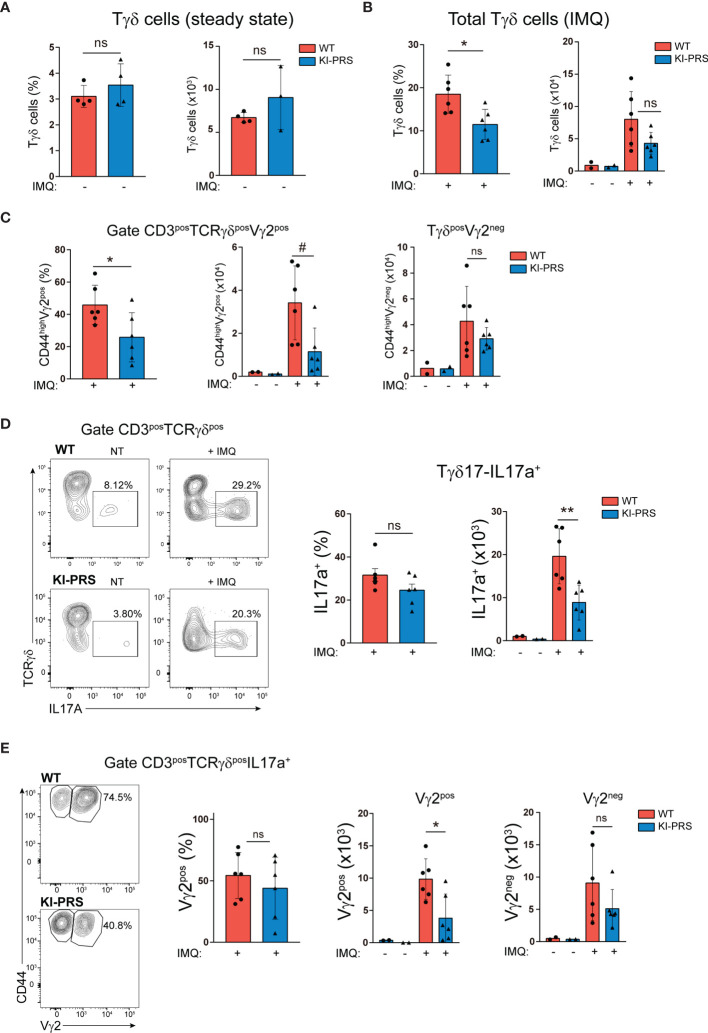
Mutation of the CD3ε-PRS reduces IMQ-induced Tγδ skin infiltration. KI-PRS and WT littermates were treated with Imiquimod (IMQ) for 7 days on ears and shaved backs, or left untreated (NT). On day 7, ears were processed for the analysis of Tγδ infiltration by flow cytometry. **(A)** Graphs represent the frequency (*left*) and absolute cell number (*right*) of total Tγδ cells in the skin of untreated mice, gated as CD3^pos^TCRγδ^pos^. **(B)** Graphs represent the frequency (*left*, *p-value= 0.0152) and absolute cell number (*right graph*) of total Tγδ cells in the inflamed skin, gated as CD3^pos^TCRγδ^pos^. **(C)** Graphs represent the frequency (*left*, *p-value= 0.0411) and absolute cell number (*right*, ^#^p-value= 0.0152) of Vγ2^pos^ and Vγ2^neg^ cells among total Tγδ cells, gated as CD3^pos^TCRγδ^pos^. **(D)** Analysis of IL-17A production. Cell suspensions obtained from inflamed and non-treated skin were stimulated with PDBu/Io for 4h in presence of Golgi-Plug, and IL-17A production was determined by flow cytometry. Representative dot plots show IL-17A production among Tγδ cells (gated as CD3^pos^TCRγδ^pos^), and inset numbers represent the percentage of IL-17A+ cells. Graphs show the frequency (*left*) and absolute cell number (*right*, **p-value= 0.0043) of IL-17A-producers among Tγδ cells. **(E)** Representative dot plots show Vγ2 expression among IL-17A-producing Tγδ cells (gated as TCRγδ^pos^IL-17A+). Graphs represent the frequency (*left*) and absolute cell number (*right*, *p-value= 0.0260) of Vγ2^pos^ and Vγ2^neg^ cells among IL-17A-producing Tγδ cells. All graphs represent mean ± sd of n=6 mice of each genotype. Statistical analysis was performed using Mann-Whitney t-test. ns, non significant. Data are representative of 2 independent experiments.

The effector function of Tγδ17 cells is required for the development of Imiquimod (IMQ) skin inflammation model (29, 30). In this psoriasis-like model, Tγδ17 cells expand in the LN and then migrate to the inflamed skin (30, 32, 33), where they contribute to the development of the skin lesions through the secretion of IL-17A (29, 34, 35). To determine if Tγδ17 pathogenic function was altered in KI-PRS mice, we first assessed the formation of psoriatic-like lesions was altered in the IMQ skin inflammation model. KI-PRS and WT littermates were treated with IMQ for 7 days, and back skin sections were subjected to H&E staining (**Figure 4A** and **Supplemental Figure S1**). The epidermal thickness was quantified at multiple sections and sites, randomly chosen in a blind manner. In two independent experiments, individual measures showed a significant attenuation of IMQ-induced epidermis thickening in KI-PRS mice compared to their WT littermates (**Figure 4B**). The reduction in epidermis thickening was also significant when the data was plotted as an average value in an individual mouse basis (**Figure 4C**). Next, we explored if the reduction in epidermal function in KI-PRS mice was accompanied by a decreased leucocyte infiltrate. In the steady state, we found that both the frequency and absolute cell number of Tγδ cells were normal in KI-PRS mice compared to WT littermates (**Figure 5A**). However, upon IMQ challenge, there was a significant reduction in the frequency and absolute cell number of total Tγδ cells (**Figure 5B**). In the IMQ skin-inflammation model, dermal Tγδ-Vγ2 cells are main source of IL-17 and require extrathymic differentiation (8, 30). The analysis of skin infiltrated Tγδ-Vγ2^pos^ cells showed a marked and significant decrease of this cell population in KI-PRS mice (**Figure 5C** **).** Full gating strategy for skin Tγδ cells in shown in **Supplemental Figure S2**. The population of Tγδ-Vγ2^neg^ was also reduced in KI-PRS mice, although the data did not reach statistical significance (**Figure 5C**). As the development of the skin lesions in the IMQ model partially depends on the secretion of IL-17A by Tγδ17 cells (29, 35), we measured the number of IL-17A-producing Tγδ cells in the inflamed skin and found approximately a 50% reduction in KI-PRS mice compared to WT controls (**Figure 5D**). In addition, we assessed the usage of Vγ2 rearrangement among IL-17A producers. Although the frequency of Vγ2 cells among IL-17A producers was not altered, the absolute cell number of Tγδ^pos^IL-17^pos^Vγ2^pos^ cells showed a marked and significant decrease in KI-PRS mice. Skin-infiltrated Tγδ^pos^IL-17^pos^Vγ2^neg^ cells were also slightly diminished, although the data did not reach statistical significance (**Figure 5E**). The IMQ model induces skin myeloid cell infiltration that resemble human psoriasis (31). We found no differences in absolute cell number of total myeloid cells (CD11b^pos^), recruited monocyte-macrophages (CD11b^pos^Ly6C^pos^Ly6G^neg^) or neutrophils (CD11b^pos^Ly6C^neg^Ly6G^pos^) in IMQ-treated KI-PRS mice vs WT controls (**Supplemental Figures S3**, **S4**). Thus, the mutation in the CD3ε-PRS sequence did not have a global impact on the infiltration of myeloid cells in the skin, in spite the fact that epidermis engrossment caused by IMQ was clearly ameliorated (**Figure 4**). Thus, the effect of the CD3ε-PRS mutation on skin thickening could be explained by a defect in the generation and recruitment of Tγδ17 cells to the skin that is not accompanied by a deficient recruitment of myeloid inflammatory cells.

### Lymph Node Tγδ17 Expansion in Imiquimod-Induced Skin Inflammation Depends on CD3ε-PRS

**Figure 6:**
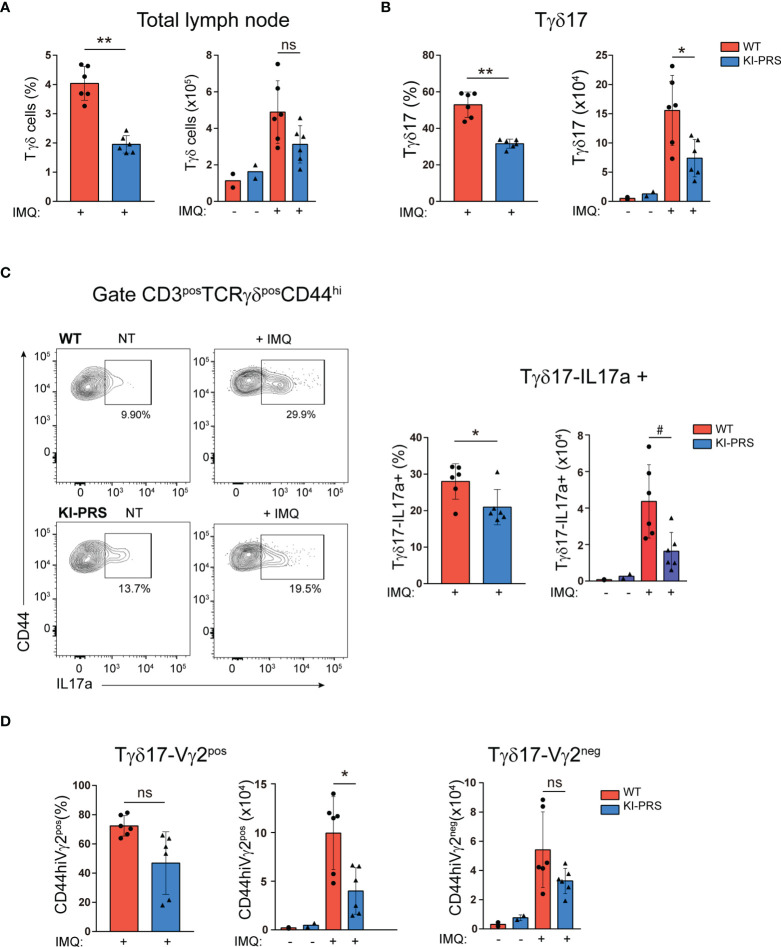
Lymph node Tγδ17 expansion upon Imiquimod-induced skin inflammation challenge depends on an intact CD3ε-PRS. KI-PRS and WT littermates were treated with Imiquimod (IMQ) for 7 days on ears and shaved backs, or left untreated (NT). On day 7, skin-draining lymph nodes were harvested and processed for flow cytometry analysis of Tγδ cells. **(A)** Graphs represent the frequency (*left*, **p-value= 0.0022) and absolute cell number (*right*) of total Tγδ cells gated as CD3^pos^TCRγδ^pos^. **(B)** Graphs represent the frequency (*left*, **p-value= 0.0022) and absolute cell number (*right*, *p-value= 0.0411) of Tγδ17 cells, gated as CD3^pos^TCRγδ^pos^CD44^hi^. **(C)** Analysis of IL-17A production. Total lymph node cells were stimulated with PDBu/Io for 4h in presence of Golgi-Plug, and IL-17A production was determined by flow cytometry. Representative dot plots show IL-17A production among Tγδ17 cells (gated as CD3^pos^TCRγδ^pos^CD44^hi^), and inset numbers represent the percentage of IL-17A+ cells. Graphs show the frequency (*left*, *p-value= 0.0312) and absolute cell number (*right*, ^#^p-value= 0.0152) of IL-17A-producers among Tγδ17; cells. **(D)** Graphs represent the frequency (*left*) and absolute cell number (*right*, *p-value= 0.0411) of Vγ2^pos^ and Vγ2^neg^ cells among Tγδ17 cells, gated as CD3^pos^TCRγδ^pos^CD44^hi^. All graphs represent mean ± sd of n=6 mice of each genotype. Statistical analysis was performed using Mann-Whitney t-test. ns, non significant. Data are representative of 2 independent experiments.

In the IMQ model, Tγδ17 cells expand in the LN, and then migrate to the inflamed skin (30, 32, 33), where they contribute to development of the skin lesions. We found that epidermal thickness and the skin infiltration of IL-17-producing Tγδ cells upon IMQ treatment was reduced in KI-PRS mice (**Figures 4**, **5**). Therefore, we next investigated if Tγδ17 expansion in the LN was affected in KI-PRS mice. We treated KI-PRS and WT littermates with IMQ for 7 days and measured the expansion of Tγδ17 cells in the LN (**Figure 6**). The frequency of total Tγδ cells was significantly reduced in KI-PRS mice, and the absolute cell number was reduced although the difference did not reach statistic significance (**Figure 6A**). The specific analysis of Tγδ17 cells showed approximately a 50% decrease both in percentage and absolute cell number (**Figure 6B**). To determine the functionality of IMQ-induced Tγδ17 cells, we determined their ability to produce IL-17A upon stimulation with phorbol ester and ionomycin. These experiments showed that KI-PRS Tγδ17 population comprised a lower frequency of IL-17A producing cells compared to their WT littermates (**Figure 6C**), suggesting that in addition to the reduction in cell number, the pathogenic function of KI-PRS Tγδ17 cells was also impaired in the IMQ model. The usage of Vγ2 rearrangement is highly frequent among IMQ-induced Tγδ17 cells (30, 32). Thus, consequently with the deficit in this population detected in the thymus and LN of untreated mice, the analysis of Vγ2 showed that the frequency and absolute cell number of Tγδ17-Vγ2^pos^ cells were strongly decreased in IMQ-treated KI-PRS mice (**Figure 6D**). Overall, **Figure 6** shows that the generation and expansion of pathogenic Tγδ17 in the LN upon IMQ challenge strongly depend on the presence of an intact CD3ε-PRS sequence. To summarize, our results on KI-PRS mice reveal that specific signaling pathways downstream the TCR are required for optimal Tγδ differentiation in the thymus. Moreover, we concluded that an intact CD3ε-PRS is required for maximal pathogenic function of Tγδ17 cells and thus, the interference with the TCR signaling emanating from CD3ε-PRS may offer a novel therapeutic opportunity for treatment of Tγδ17-mediated diseases.

## Discussion

The role of TCR signaling during the intrathymic commitment of Tγδ cells towards the Tγδ17 lineage has not been completely elucidated. Seminal work suggested that Tγδ differentiation requires a quantitatively different TCR signaling: strong TCR signaling leads to commitment towards IFNγ secretion, while Tγδ17 differentiation requires weak or no TCR signals. In contrast, several studies in animals with null or altered expression of TCR-proximal kinases have shown that attenuated TCR signaling causes a specific impairment of Tγδ17 cell differentiation. Those data suggest that the requirements of TCR signaling for commitment towards the Tγδ17 lineage might not only be quantitative but also qualitative. Here, we report that mice bearing two conservative point mutations in the polyproline sequence of CD3ε (PxxP to AxxA, KI-PRS mice) that disrupt the binding site for the adaptor protein Nck have an impaired commitment towards Tγδ17 lineage in the thymus, accompanied by decreased frequency and absolute cell number of Tγδ17 cells in the LN. Furthermore, we have addressed the pathogenic function of Tγδ17 cells in the Imiquimod model of skin inflammation, and determined that KI-PRS mice presented attenuated epidermis engrossment, and impaired generation, expansion and effector function of Tγδ17 cells in KI-PRS mice compared to WT littermates. Therefore, we conclude that an intact CD3ε-PRS sequence is required for optimal differentiation and pathogenic function of Tγδ17 cells, revealing a specific TCR signaling dependence for development and function of these pro-inflammatory cells.

Our results on KI-PRS support the idea that a unique configuration of the TCR signalosome dictates Tγδ cell commitment. Other scientific evidences also support the existence of a distinct/unique TCR signalosomes in the different Tγδ subpopulations. For example, RNAseq data available from the Immunological genome project (www.immgen.org) (45) of Tγδ17 vs Tγδ-IFNγ subpopulations (Tgd.g2posd17.LN vs Tgd.g2posd1.LN datasets) show a 20fold decrease in Lck mRNA expression in Tγδ17 and in contrast, a 13fold increase in the expression of the Src kinase family member Blk compared to Tγδ-IFNγ cells. These differences, albeit less prominent, are also present in thymic Tγδ subpopulations. Accordingly, Tγδ17 differentiation is strongly reduced in Blk-deficient animals (21). RNAseq data do not show major differences in the expression of other proximal TCR signaling components such as Fyn, ZAP70 or Syk, although altered expression or function of both ZAP70 and Syk have been show to impair Tγδ17 differentiation (22–24). All considered, the data suggest that the requirements of TCR signaling for Tγδ17 differentiation are not only quantitative but also qualitative.

The notion that a unique configuration of the TCR signalosome is required for Tγδ17 development assumes that the defects observed in KI-PRS mice are cell-intrinsic, which is supported by some available data. For example, the absolute number of thymocytes is not altered in KI-PRS mice (37). Thus, it is not likely that the developmental impairment observed in KI-PRS mice is a consequence of increased homeostatic proliferation of TCRγδ cell to fill the thymus niche. Additionally, we have not observed differences in absolute cell number of immature TCRγδ progenitors (CD24^pos^CD73^neg^), suggesting that the input of cells up to this developmental stage is normal, and that the impairment is restricted to further developmental stages. However, we cannot formally exclude the possibility that the differentiation of TCRγδ cell in the thymus of KI-PRS mice is influenced by differences in the conventional TCRαβ cell compartment that also carry the CD3ε-PRS mutation. Further research will be required to establish and validate *in vitro* assays that recapitulate all the stages of Tγδ17 cell development in the thymus and generate bona-fide, mature Tγδ17 cells. This type of experiments will clearly determine if defects in KI-PRS Tγδ17 cells are cell-intrinsic.

The analysis of Tγδ17 differentiation in the thymus show that the impairment in KI-PRS mice occurs in the most immature stage (CD24^pos^CD73^neg^), where KI-PRS Tγδ17 progenitors down-regulate CD27 and induce the expression of RORγt, but they fail to up-regulate CD44 and to down-regulate CD45RB. Thus, there is an inefficient progression to the subsequent developmental stages that finally cause a reduction in the absolute cell number of mature Tγδ17 cells. These experiments suggest that the TCR signaling emanating from the CD3ε-PRS is not essential for the expression of the transcription factor RORγt, but they are required for further progression of Tγδ17 progenitors towards mature stages (CD44^hi^CD45RB^neg^). The deffects observed in KI-PRS mice are likely to be mediated by the adaptor protein Nck. Nck is a SH2/SH3 adaptor protein that plays a pivotal role in coordinating the signaling networks critical for organizing the actin cytoskeleton, cell movement, adhesion, or axon guidance, and for connecting transmembrane receptors to multiple intracellular signaling pathways (46–48). Nck is directly recruited to the CD3ε-PRS upon TCR triggering *via* its N-terminal SH3 (SH3.1) domain (49), and the conservative mutations on polyproline sequence of CD3ε (PxxP to AxxA) disrupts the binding of the adaptor protein Nck upon TCR triggering (37). In TCRαβ cell thymic differentiation, the KI-PRS mice presented an impairment in thymic development at the stages were pre-TCR or TCR signaling was required (37). In mature TCRαβ cells, CD3ε-PRS mutation caused a partial reduction of TCR-proximal activation events such as CD3ζ and ZAP70 phosphorylation, and decreased ZAP‐70 recruitment to the TCR-CD3 complex. These TCR-proximal effects are paralleled by decreased TCR-induced proliferation and spreading, and impaired effector function of both CD8 and CD4 T cells (38). Mechanistically, it has been recently found that Nck is required for Lck recruitment to the upon stimulation for optimal TCR signaling (50). As mentioned above, Blk and not Lck is the major Src kinase family member in Tγδ17 cells. Further research will be required to determine if Nck recruitment to CD3ε-PRS is also required for Blk binding and activation in Tγδ17 cells. Nck downstream effectors include proteins involved in actin cytoskeleton reorganization such as the SCAR/WAVE proteins or the serine-threonine kinase Pak1 (51) or critical components of the TCR signaling machinery such as SLP76 (52, 53). All these data have been generated in TCRαβ cells, and further work will be required to determine if these CD3ε-PRS downstream events are conserved in Tγδ cells. Interestingly, Nck main function is closely related to the regulation of actin cytoskeleton remodeling and so far, the role of TCR-regulated actomyosin contractile networks in Tγδ17 differentiation or effector function has not been addressed, although actomyosin cytoskeleton reorganization is required for Tγδ17 and Th17 migration to the inflamed site in the IMQ model of psoriasis (54). Thus, further research is required to fully characterize the critical components of the Tγδ17 TCR signalosome. Interestingly, this new knowledge can generate novel therapeutic opportunities for treatment of Tγδ17-mediated autoimmune diseases. In this context, AX-024 is an orally available, low-molecular weight inhibitor of CD3ε-Nck protein-protein interaction (55). Remarkably, administration of AX-024 exerted therapeutic benefits in IMQ-induced skin inflammation, in OVA-induced allergic asthma and in experimental autoimmune encephalomyelitis (multiple sclerosis model). *In vitro* treatment of Tαβ cells with AX-024 was found to attenuate TCR proximal signaling events, TCR-induced T cell proliferation and to impair differentiation towards pro-inflammatory effector cells (Th1, Th17) while favoring regulatory T cell (Tregs) generation. In this context, it will be interesting for further research to study the effects of AX-024 on Tγδ17 cells to demonstrate the implication of Nck binding to CD3ε in differentiation and pathogenic function of this subpopulation. If the administration of AX-024 can impair the generation of Tγδ17 cells in the IMQ model, this drug has a great potential not only as preventive but also as curative effect. In addition to the Tγδ17, Tregs and granulocyte-macrophage colony-stimulating factor (GM-CSF)-producing CD4^pos^ T cells are also involved in the development of the IMQ skin inflammation model, where Tregs restrain the skin infiltration of pathogenic (GM-CSF)-producing CD4^pos^ T cells (56). Thus, the protective effects of AX-024 observed in the IMQ model maybe also mediated by reduced generation of pro-inflammatory CD4 T cells and increased numbers of Tregs (55). Further research will be required to determine if AX-024 protective effects are mediated by Tγδ17, Tαβ, or both cell types.

IMQ challenge induces *de novo* generation and expansion of Tγδ17 cells (8) that then migrate to the inflamed skin to exert their pathogenic effector function (30, 32, 33). Interestingly, IMQ-induced Tγδ17 cells are endowed with memory-like features such as long-term survival and the ability to mount faster and greater responses to a second IMQ challenge (33, 36). These memory-like characteristics maybe related to the relapsing/remitting feature of inflammatory pathologies such as psoriasis or multiple sclerosis. Thus, the interference with Tγδ17 cells with the AX-024 or other specific inhibitors during the first challenge has the potential to reduce the generation of Tγδ17 memory cells and thus, to ameliorate clinical symptoms during psoriasis flares.

We have observed that the frequency and absolute cell number of Vγ2 cells among uncommitted, TCRγδCD44^low^ subpopulation were reduced in KI-PRS mice both in thymus and LN. Thus, it remains an open question if an intact CD3ε-PRS is required specifically for the generation of all Vγ2 cells in the thymus, or only for specific subpopulations. In this context, we found that the number of Tγδ-IFNγ cells using the Vγ2 rearrangement was normal, suggesting that CD3ε-PRS is required for unique Tγδ effector subsets rather than for all Vγ2 cells. As mentioned above, the number of Tγδ-IFNγ cells was not altered in the LN of KI-PRS mice, demonstrating that this particular Tγδ subset did not require CD3ε-PRS signaling for its generation. However, we have not addressed if the effector function of Tγδ-IFNγ is altered in these animals using tumor models (3). Interestingly, it has been shown that cytotoxic human Tγδ cells did not require Nck recruitment to CD3ε to exert their tumor killing activity (57). Cytotoxic human Tγδ cells have the ability to produce IFNγ (3, 58) and thus, the available data suggest that Tγδ-IFNγ do not require Nck binding to CD3ε-PRS to exert their effector function.

In summary, we show that the polyproline sequence of CD3ε is required for Tγδ17 commitment in the thymus, and for the expansion and exertion of pathogenic function of this Tγδ subpopulation in IMQ-induced skin inflammation model. These results support the idea that TCR signaling requirements for Tγδ17 differentiation are not only quantitative but also qualitative, and that a unique arrangement of the TCR signalosome dictates Tγδ cell commitment and effector function. Although further research is required to fully characterize the critical components of the Tγδ17 TCR signalosome, this notion opens new interesting opportunities for specific therapeutic intervention in Tγδ17 mediated autoimmune and inflammatory diseases.

## Data Availability Statement

The original contributions presented in the study are included in the article/**Supplementary Material**. Further inquiries can be directed to the corresponding author.

## Ethics Statement

The animal study was reviewed and approved by Comunidad de Madrid PROEX-296-7-21.

## Author Contributions

AB carried out experimental work, analyzed data, prepared Figures and edited the manuscript, BA supervised the work, provided resources and edited the manuscript, MN carried out experimental work, supervised the experiments, analyzed data, provided resources and wrote the manuscript. All authors contributed to the article and approved the submitted version.

## Funding

This work was supported by grants PID2019-110511RB-I00 to M.N.N and PID2019-104935RB-I00 to B.A, funded by the Spanish Ministry of Science and Innovation (Ministerio de Ciencia e Innovación, MCIN) and Agencia Estatal de Investigación (AEI) MCIN/AEI/10.13039/501100011033/.

## Conflict of Interest

The authors declare that the research was conducted in the absence of any commercial or financial relationships that could be construed as a potential conflict of interest.

## Publisher’s Note

All claims expressed in this article are solely those of the authors and do not necessarily represent those of their affiliated organizations, or those of the publisher, the editors and the reviewers. Any product that may be evaluated in this article, or claim that may be made by its manufacturer, is not guaranteed or endorsed by the publisher.

## Supplementary Material

Supplementary Figure 1 Attenuation of IMQ-induced psoriasis-like lesions in KI-PRS mice. KI-PRS and WT littermates were treated with Imiquimod (IMQ) for 7 days on ears and shaved backs, or left untreated (NT). On day 7, back skin sections were subjected to hematoxylin and eosin (H/E) staining for microscopy analysis. The thickness of the epidermal layer was measured at multiple sections and sites, randomly chosen in a blind manner. The figure shows representative sections of H/E staining in the indicated conditions.

Supplementary Figure 2 Gating strategy for skin TCRγδ analysis. Representative dot plots show full gating strategy for skin TCRγδ analysis.

Supplementary Figure 3 Gating strategy for skin myeloid infiltrate. Representative dot plots show full gating strategy for skin myeloid infiltrate analysis.

Supplementary Figure 4 IMQ-induced myeloid infiltrate in skin. KI-PRS and WT littermates were treated with Imiquimod (IMQ) for 7 days on ears and shaved backs, or left untreated (NT). On day 7, ears were processed for the analysis of myeloid cell infiltration by flow cytometry. Graphs represent the frequency *(left)* and absolute cell number *(right)* of the indicated cell populations. **(B)** CD45.2^pos^ cells. **(C)** Myeloid cells, gated as CD11b^pos^. **(D)** Dendritic cells, gated as CD11c^pos^. **(E)** Neutrophils, gated as CD11b^pos^Ly6C^neg^Ly6G^pos^. **(F)** Inflammatory monocyte-macrophages, gated as CD11b^pos^Ly6C^pos^Ly6G^neg^. **(G)** Resident monocytes, gated as CD11b^pos^Ly6C^neg^Ly6G^neg^. Statistical analysis was performed using Mann-Whitney t-test. ns, non significant. Data are representative of 2 independent experiments.
