# Supplementary figures and images for: Mutation of the Polyproline Sequence in CD3ε Evidences TCR Signaling Requirements for Differentiation and Function of Pro-Inflammatory Tγδ17 Cells
Source: Front Immunol. 2022 Mar 31;13:799919. doi: 10.3389/fimmu.2022.799919 (PMC9008450; doi:10.3389/fimmu.2022.799919)

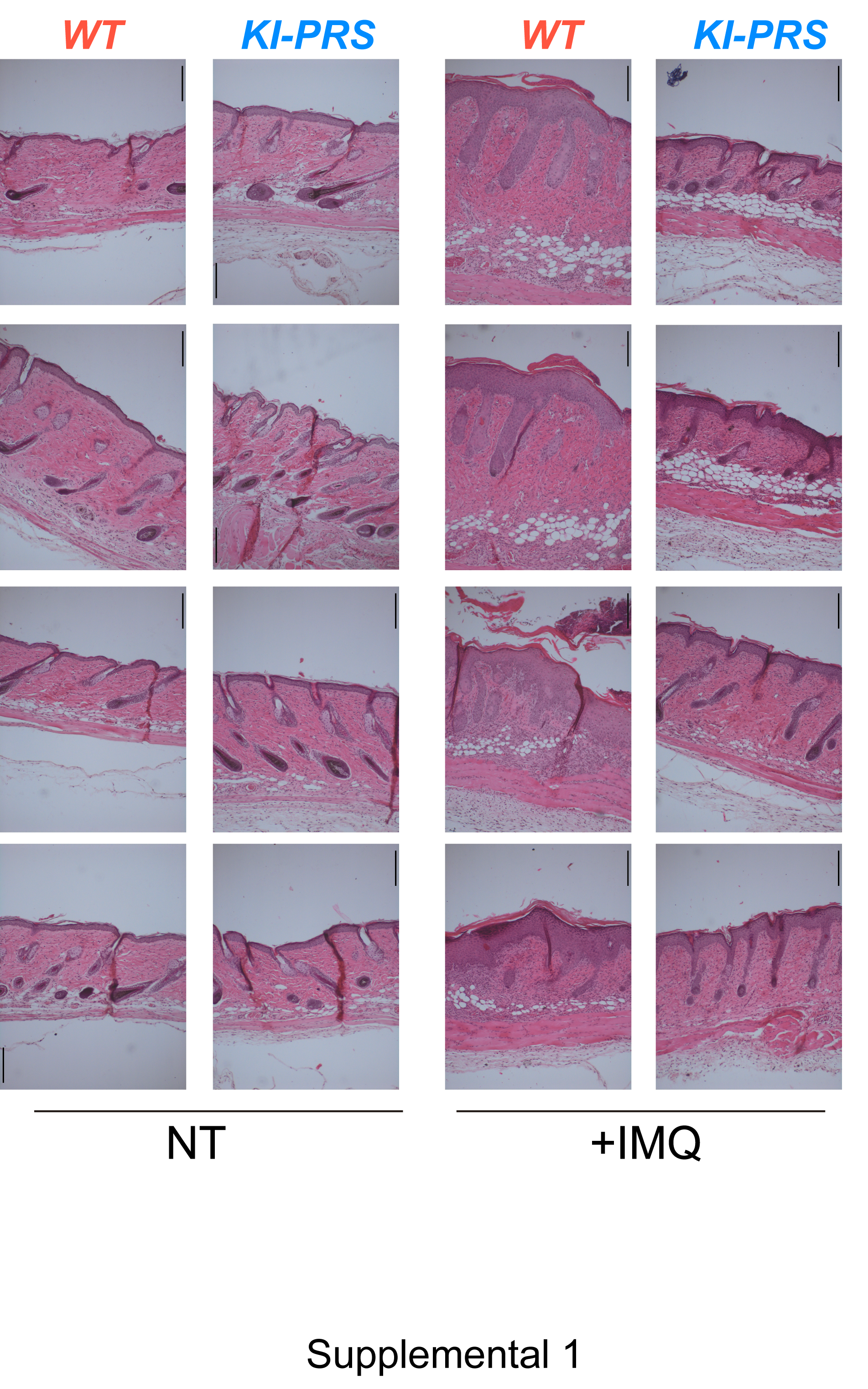

Supplement: Supplementary Figure 1 — Attenuation of IMQ-induced psoriasis-like lesions in KI-PRS mice. KI-PRS and WT littermates were treated with Imiquimod (IMQ) for 7 days on ears and shaved backs, or left untreated (NT). On day 7, back skin sections were subjected to hematoxylin and eosin (H/E) staining for microscopy analysis. The thickness of the epidermal layer was measured at multiple sections and sites, randomly chosen in a blind manner. The figure shows representative sections of H/E staining in the indicated conditions. [file Image_1.tif]

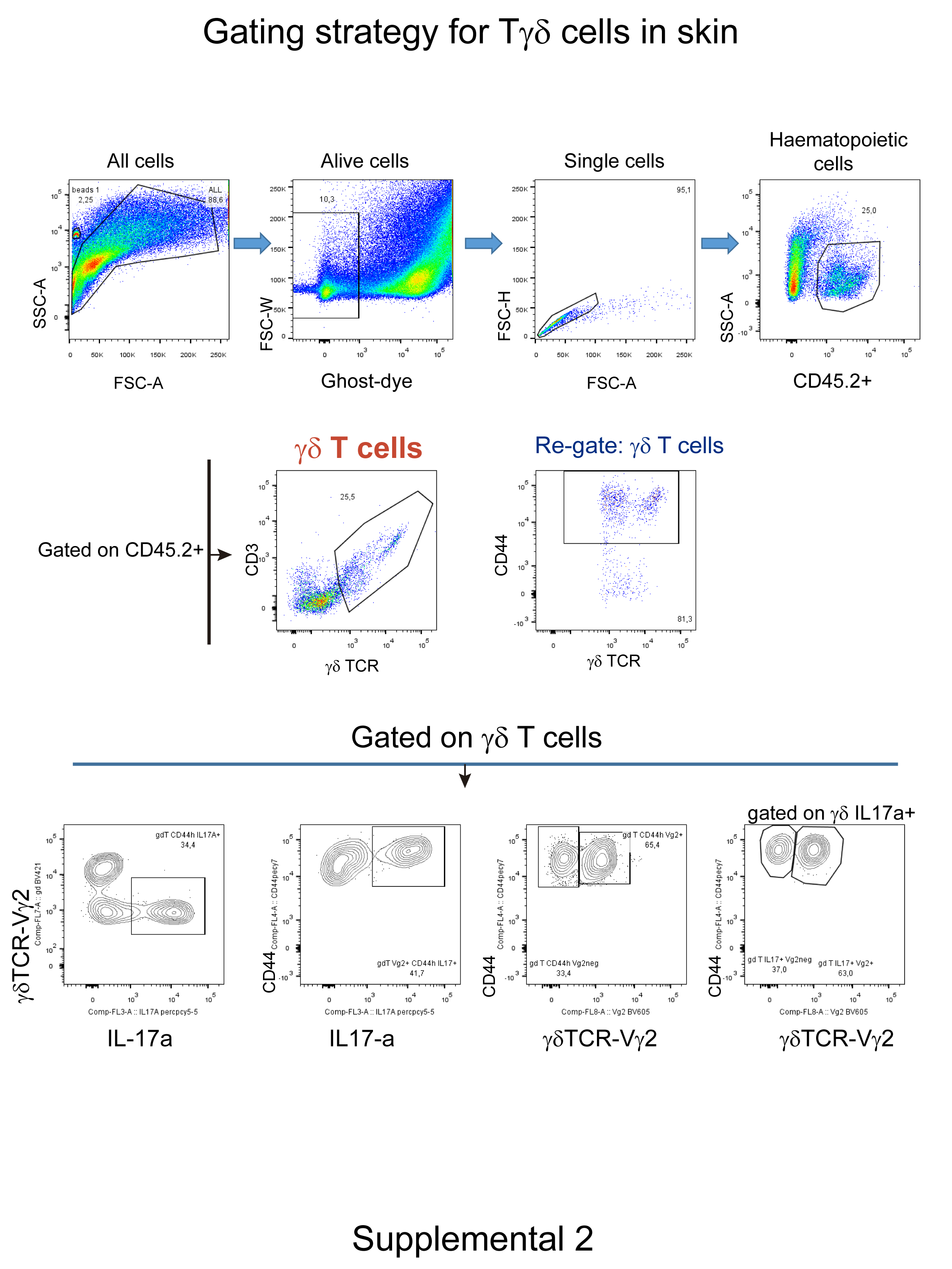

Supplement: Supplementary Figure 2 — Gating strategy for skin TCRγδ analysis. Representative dot plots show full gating strategy for skin TCRγδ analysis. [file Image_2.tif]

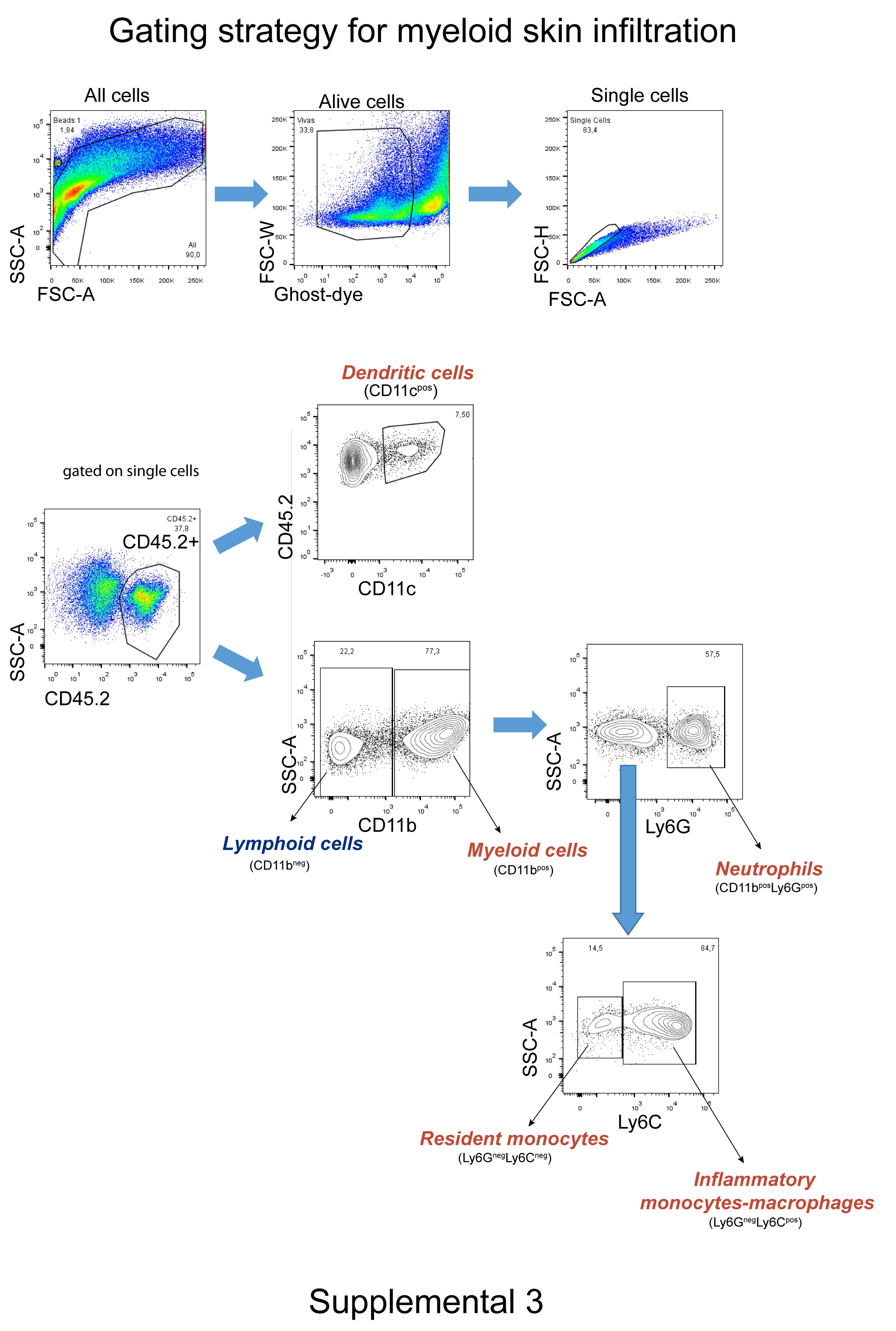

Supplement: Supplementary Figure 3 — Gating strategy for skin myeloid infiltrate. Representative dot plots show full gating strategy for skin myeloid infiltrate analysis. [file Image_3.tif]

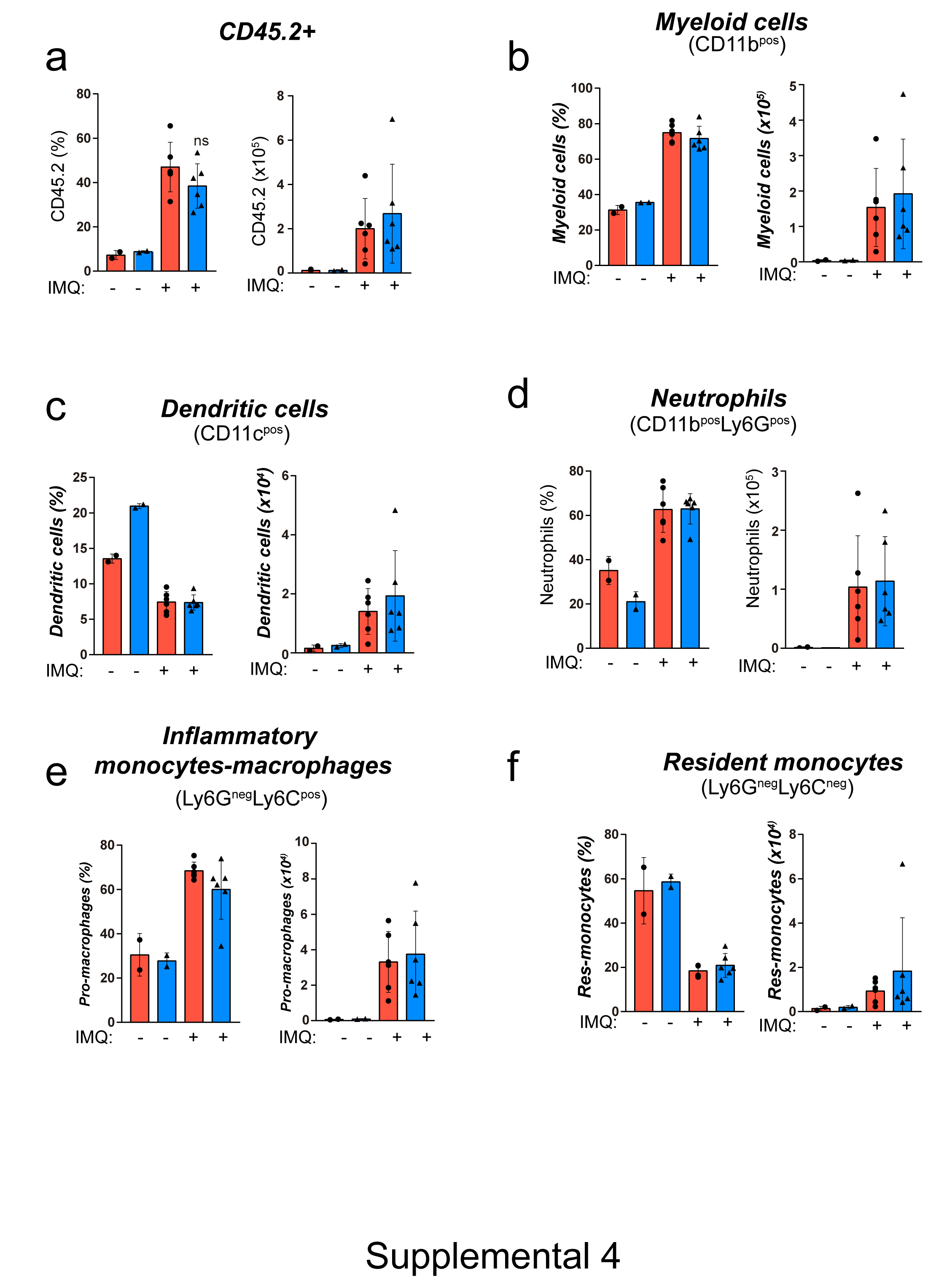

Supplement: Supplementary Figure 4 — IMQ-induced myeloid infiltrate in skin. KI-PRS and WT littermates were treated with Imiquimod (IMQ) for 7 days on ears and shaved backs, or left untreated (NT). On day 7, ears were processed for the analysis of myeloid cell infiltration by flow cytometry. Graphs represent the frequency (left) and absolute cell number (right) of the indicated cell populations. (B) CD45.2pos cells. (C) Myeloid cells, gated as CD11bpos. (D) Dendritic cells, gated as CD11cpos. (E) Neutrophils, gated as CD11bposLy6CnegLy6Gpos. (F) Inflammatory monocyte-macrophages, gated as CD11bposLy6CposLy6Gneg. (G) Resident monocytes, gated as CD11bposLy6CnegLy6Gneg. Statistical analysis was performed using Mann-Whitney t-test. ns, non significant. Data are representative of 2 independent experiments. [file Image_4.tif]
